# Disentangling the Effects of Biotic and Abiotic Dimensions of Ecological Opportunity on Individual Trophic Trait Variation

**DOI:** 10.1111/mec.70115

**Published:** 2025-09-24

**Authors:** Kurt Villsen, Gaït Archambaud‐Suard, Emese Meglécz, Simon Blanchet, Jean‐Pierre Balmain, Mathilde Bertrand, Rémi Chappaz, Vincent Dubut, Emmanuel Corse

**Affiliations:** ^1^ Aix Marseille Univ, Avignon Université, CNRS, IRD, IMBE Marseille France; ^2^ INRAE, Aix Marseille Univ, RECOVER Aix‐en‐Provence France; ^3^ CNRS, Station d'Écologie Théorique et Expérimentale (UAR 2029–SETE) Moulis France; ^4^ ADENEKO Saint‐Girons France; ^5^ Université de Mayotte Dembeni France; ^6^ MARBEC, CNRS, Ifremer, IRD, Université de Montpellier Montpellier France

**Keywords:** conservation, diet metabarcoding, ecological opportunity, individual niche variation, path analysis

## Abstract

Within‐species individual trait variation (ITV) plays a critical role in ecological and evolutionary dynamics by influencing community structure, ecosystem functioning, and individual fitness. While the role of the biotic dimension of ecological opportunity (e.g., interspecific competition, prey availability) in shaping trophic ITV is now well established, the role of the abiotic dimension, and its interactions with biotic factors, remains critically overlooked, limiting our understanding of how individuals cope with changes in ecological opportunity. To address this knowledge gap, we investigated trophic ITV in the endangered riverine fish *Zingel asper* using a multi‐faceted approach: (i) precise quantification of trophic ITV via faecal metabarcoding, (ii) fine‐scale mapping of prey availability and habitat structure across seasons, and (iii) path analysis to assess the direct and indirect effects of prey, habitat, and their spatial heterogeneity in driving ITV. The individual niche width (INW) in *Z. asper* was largely determined by preferred prey availability, while between‐individual variation (BIC) was largely determined by a combination of prey and habitat factors, with habitat exerting a direct effect (i.e., not mediated via prey) on trophic ITV. This study provides a mechanistic explanation of the processes underlying the shift from selective to opportunistic foraging strategies. Notably, we demonstrated that trophic ITV is altered by the interaction between predator life‐history traits (in the present study, size) and four distinct dimensions of ecological opportunity: prey availability and their spatial distribution, habitat structure and seasonality.

## 1 Introduction

Understanding the ecological drivers of trophic niche variation is a central aim of trophic ecology, providing insights into predator–prey interactions, food‐web structure, and the evolution of biodiversity (Bolnick et al. 2011; Brodersen et al. 2018; Schreiber et al. 2011). The trophic niche was long thought to vary primarily at the population or species level, with individuals considered to be ecologically equivalent (Bolnick et al. 2003; MacArthur and Levins 1967). However, Individual Trait Variation (ITV) is now recognised as a key driver of ecological and evolutionary dynamics (see Gamelon et al. 2025) and as an important source of functional diversity (Des Roches et al. 2018; Raffard et al. 2019, 2021). The trophic component of ITV reflects individual strategies to optimise food acquisition (Roughgarden 1972) and can be quantified using two individual trophic traits: the between‐individual component (BIC; related to β‐diversity and individual specialisation) and the individual niche width (INW; associated with α‐diversity) (Roughgarden 1974). Trophic ITV is known to influence the outcome of ecological interactions (e.g., predator–prey or competitive interactions; Bolnick et al. 2011; Hart et al. 2016) by promoting variation in interaction strength among individuals that differs from the population mean interaction strength (i.e., Jenson's inequality; Ruel and Ayres 1999). The degree of trophic ITV within populations may also reflect resilience to disturbance, wherein populations with homogeneous foraging strategies may be more sensitive to changes in resource availability (Costa‐Pereira, Toscano, et al. 2019; MacColl 2011). A better understanding of the factors that drive trophic ITV may therefore improve our capacity to predict how trophic interactions and species will respond under diverse future scenarios (e.g., global change; Gamelon et al. 2025; Singh et al. 2024; Urban et al. 2016).

Trophic ITV is known to vary according to ecological opportunity (Roughgarden 1972; Araújo et al. 2011), that is: the biotic and abiotic niche space that can be exploited at a given moment by an individual, a population, or a species (Araújo et al. 2011; Evangelista et al. 2014; Sjödin et al. 2018). Past research has highlighted the importance of the biotic dimension of ecological opportunity (prey abundance and diversity, ontogenic shifts, competition, and predation risks) in driving trophic ITV (Bolnick and Ballare 2020; Costa‐Pereira, Araújo, et al. 2019; Sánchez‐Hernández et al. 2019, 2021). In contrast, although habitat conditions are known to modify foraging behaviour, predator selectivity, and energetic foraging costs (Bartels et al. 2012; Champion et al. 2018; Facey and Grossman 1992; Wood et al. 2013), the abiotic drivers of trophic ITV remain poorly understood. In addition to abiotic conditions, the spatial dimension of ecological opportunity is rarely considered in studies of trophic ITV (but see: Walker et al. 2023). Spatial heterogeneity is expected to strongly influence consumer access to resources (MacArthur and Pianka 1966; Stephens and Krebs 1986) and may therefore have a significant effect on trophic ITV (Bolnick and Ballare 2020; Trevail et al. 2021; Walker et al. 2023). Indeed, the spatial distribution of resources can strongly influence the opportunity cost of stopping to consume a resource rather than continuing the search for preferred resources (MacArthur and Pianka 1966; Stephens and Krebs 1986). In accordance with this theory, several empirical studies have demonstrated that spatial resource heterogeneity can also translate to spatial variation in individual diets (Mikheev et al. 2010; Walker et al. 2023). However, to the best of our knowledge, no study has addressed how spatial heterogeneity in both biotic and abiotic dimensions of ecological opportunity jointly drives trophic ITV.

A major challenge in studying the effects of the abiotic dimension of ecological opportunity on trophic ITV is that the biotic and abiotic dimensions are not independent. For example, abiotic conditions can affect both prey availability (i.e., spatial and temporal distributions) and predator trophic ITV (e.g., by modifying predator foraging behaviour; Bartels et al. 2012; Musseau et al. 2015). Disentangling direct abiotic effects from indirect, prey‐mediated effects on individual niche variation therefore necessitates evaluating the separate and combined effects of biotic and abiotic factors on trophic ITV (Costa‐Pereira and Shaner 2025). To this end, causal analysis (such as path analyses; Grace 2006; Siegel and Dee 2025; Shipley 2009) coupled with a fine‐scale description of both biotic and abiotic conditions offers a promising approach to disentangle prey and habitat effects on trophic ITV. A second challenge in studying the drivers of trophic ITV is in acquiring precise measurements of predator diet and an equivalent measurement of the ecological opportunity for an individual or population. To this end, DNA metabarcoding has emerged as a valuable tool for estimating individual trophic traits and studying inter‐ and intra‐population levels of trophic niche variation (Velarde‐Garcéz et al. 2024; Villsen, Corse, Meglécz, et al. 2022; Voelker et al. 2020). DNA‐based analyses usually provide much higher taxonomic resolution compared to other approaches, e.g., morphological analyses of stomach or faeces contents (Jakubavičiute et al. 2017; Zarzoso‐Lacoste et al. 2016). This can provide valuable additional precision especially when predators preferentially target a specific subset of species within a broader taxonomic grouping (Clare et al. 2019; Cuff et al. 2022; Villsen, Corse et al. 2025). Moreover, compared to stable isotope analyses, which are highly integrative and can recapitulate an individual's diet over several weeks or months (e.g., Novak and Tinker 2015; Petta et al. 2020), metabarcoding provides a snapshot of an individual's diet, reflecting one or a few days of feeding (Corse, Valladares, et al. 2015). Trophic traits calculated from metabarcoding data are therefore ideal for studying the fine‐scale drivers of trophic ITV, as they relate more tightly to snapshot estimates of ecological opportunity (e.g., prey availability or habitat structure).

Benthic stream fish and their riverine ecosystems may serve as a useful model system for studying the interplay between biotic, abiotic, and spatial dimensions of ecological opportunity and trophic ITV. As stated above, a major challenge in trophic ITV studies is in accurately estimating ecological opportunity, which can be particularly challenging for free‐ranging species (e.g., Trevail et al. 2021; Walker et al. 2023). However, the home range of riverine, benthic, low‐mobility fishes can be more readily approximated: (i) pelagic ecological opportunities can be disregarded, (ii) the riverbanks form the lateral boundaries of the home range, and (iii) the mean diel displacement range of the fish species can be used to define the upstream and downstream limits of the sampling area (Gerking 1959). While river ecosystems are characterised by strong interplay between biotic and abiotic conditions across spatial and temporal gradients (Poff et al. 1997; Thorp et al. 2006; Doretto et al. 2020; Perez Rocha et al. 2018), making them ideal for disentangling the abiotic and biotic drivers of trophic ITV. For example, seasonal variation in riverine macroinvertebrate assemblages (i.e., a key prey group for many fish species) is both promoted by species‐specific phenology (e.g., voltinism and seasonal emergence events; Clifford 1982; Kong et al. 2019) and varies according to local conditions, such as substrate composition (Williams and Mundie 1978), fine‐sediment load (Kaller and Hartman 2004), vegetation (Downes et al. 2000), and river flow dynamics (Villsen, Beche et al. 2025), which can change across seasons. Such temporal and spatial heterogeneity in ecological opportunity in riverine systems will likely influence the trophic ITV of riverine predators. Indeed, in a previous study, we found marked seasonal diet variation in *Zingel asper* (L. 1758), (Villsen, Corse, Meglécz et al. 2022) an endangered benthic fish, endemic to the Rhone River basin (Ford 2024). We characterised a pattern of autumnal populational niche expansion, wherein *Z. asper* exhibited higher BIC and narrower INW in autumn—a pattern of transient trophic ITV which is often associated with seasonal variation in ecological opportunity (Costa‐Pereira et al. 2017; Gerardo Herrera et al. 2008).

**FIGURE 1:**
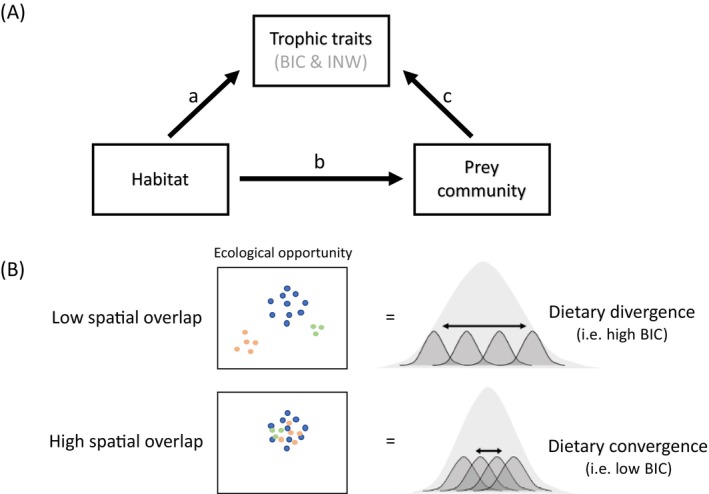
Illustration of conceptual hypotheses. (A) Confirmatory path analysis framework to be tested. Path ‘a’ relates to the direct habitat effect on individual trophic traits (INW; individual niche width and BIC; between‐individual trophic variation). The indirect habitat effect is the product of the ‘b’ and ‘c’ path estimates (i.e., estimate b * estimate c). (B) Hypothetical trophic responses to the spatial distribution of ecological opportunity (i.e., prey community and habitat factors).

The role of abiotic and spatial heterogeneity factors in how predators perceive and adapt to variation in ecological opportunity (i.e., in terms of trophic ITV) remains critically overlooked (Costa‐Pereira and Shaner 2025). As trophic ITV is expected to be an important determinant of the outcomes of species interactions (e.g., coexistence and predation pressure), it will be important to obtain a holistic understanding of the factors that drive trophic ITV across diverse taxa. We therefore aimed to understand how biotic, abiotic and spatial heterogeneity components of ecological opportunity jointly influence trophic ITV in *Z. asper* . Specifically, we tested two hypotheses: (i) trophic traits (BIC and INW) are jointly driven by biotic and abiotic factors that may directly and indirectly influence trophic ITV (i.e., a prey‐mediated habitat effect on trophic traits, see Figure 1A) and (ii) the spatial distribution of ecological opportunity (i.e., abiotic and biotic conditions) is directly related to dietary divergence (i.e., BIC; Figure 1B). To test these hypotheses, we characterised and quantified changes in ecological opportunity and trophic ITV by conducting fine‐scale sampling of prey and habitat, analysing the diet of *Z. asper* through DNA metabarcoding of faecal samples. We additionally performed prey preference analyses to better characterise the biotic dimension of ecological opportunity for *Z. asper* (i.e., identify preferred prey taxa). We then performed confirmatory path analyses to assess the direct and indirect effects of the biotic and abiotic dimensions of ecological opportunity on trophic traits (i.e., BIC and INW). As a potential additional source of inter‐individual trophic variation, we also considered the size of *Z. asper* individuals (as a proxy for ontogeny) in our path analyses. Lastly, as we previously demonstrated that *Z. asper* exhibits extensive seasonal trophic variation (Villsen, Corse, Meglécz, et al. 2022), we further investigated seasonal variation in key ecological opportunity factors to determine whether they could explain seasonal ITV in *Z. asper* .

## 2 Materials and Methods

### 2.1 Study Species: *Zingel asper*

The Rhone streber ( *Zingel asper* (L.) [Actinopterygii: Perciformes: Percidae]) inhabits small‐ to medium‐sized rocky streams from the Rhône River basin (Olivier et al. 2022). Its diet mostly consists of macroinvertebrates, but it can also occasionally consume small fish (Cavalli et al. 2003; Villsen, Corse, Meglécz, et al. 2022). *Zingel asper* also exhibits strong seasonal diet variation, with high consumption of *Baetis* and Heptageniidae mayflies in spring and summer followed by a shift to a broad range of secondary prey in autumn (e.g., Chironomidae, *Hydropsyche*, *Gammarus*; Villsen, Corse, Meglécz, et al. 2022). Individuals reach sexual maturity at 2–3 years of age and reproduce annually (Chevalier et al. 2011; Béjean 2019). Mean longevity is 4–5 years of age and adult length varies from 12 to 20 cm (Labonne and Gaudin 2005; Monnet et al. 2022). *Zingel asper* has relatively sparse population densities (from 8 to ~100 individuals ha^−1^; Villsen, Corse, Meglécz, et al. 2022), and a limited diel displacement range (50–200 m; Danancher et al. 2004; Labonne and Gaudin 2005). Its current spatial distribution is limited to four disconnected populations which represent less than 15% of its historical range (Ford 2024): one population in the Durance River basin (including the Buëch River); one in the Verdon River (a tributary of the Durance River); one in the Ardèche River basin (including the Beaume River); and one population in the Loue River (Doubs River basin). Each of these populations experiences distinct climatic conditions, varying from a semi‐continental climate in the northern range of *Z. asper* (Loue River) to Mediterranean (Ardèche River basin) and Alpine‐Mediterranean (Durance River basin and Verdon River) climates in its southern range (Olivier et al. 2022). This longitudinal gradient is associated with a trade‐off between early growth and longevity, with Loue and Ardèche populations exhibiting faster growth, larger adult size, and shorter lifespans, while the spatial pattern of growth variation is associated with variation in prey availability, hydraulics, habitat conditions, and water temperature (Monnet et al. 2022).

### 2.2 Fish and Faeces Sampling

**FIGURE 2:**
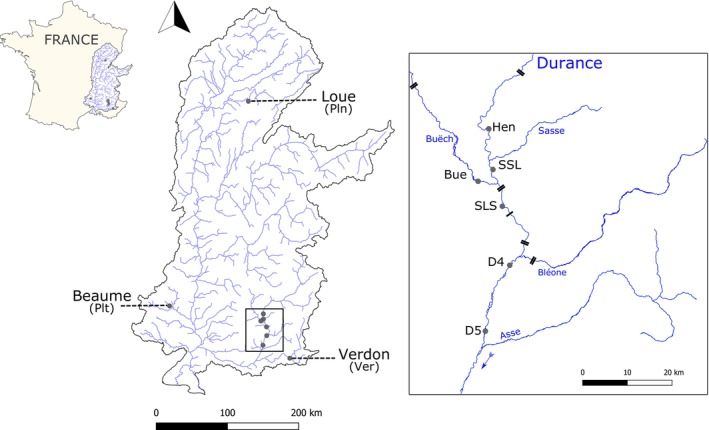
Location of sampling sites. Double and single black lines indicate dams and weirs, respectively.

*Zingel asper* faeces sampling was performed at nine sampling sites across the Rhone River basin (Figure 2). At these sites, a total of 23 sampling campaigns were performed between 2014 and 2015 in diverse seasonal conditions, i.e., autumn, summer, and spring (Table 1). In the Beaume, Loue, and Verdon rivers, fish sampling was performed at night (~02:00 to 05:00) using headlamps and dip nets (40 cm wide, 5 mm mesh net). However, this method was not applicable in the Durance and Buëch rivers due to their high flow speed and turbidity (Cavalli et al. 2003). We therefore captured fish using an electrofishing approach (200–500 V, EFKO, Germany) during the daytime. The captures were performed in accordance with permits from the French *Directions Départementales des Territoires* (DDTs) from Hautes‐Alpes, Alpes‐de‐Haute‐Provence, Ardèche, and Jura.

**TABLE 1:** Faeces metabarcoding, prey community and habitat sampling design.

Catchment area	Campaign ID	Coordinates	Campaign date	Dietary sampling size	BIC mean (SD)	INW mean (SD)	Habitat and macroinvertebrates sample size
Durance	14HenA	N 44°18′46″ E 5°55′29″	May 2014	33^a^	0.65 (0.17)	1.04 (0.69)	90
	14HenB		Oct 2014	34^a^	0.76 (0.15)	0.98 (0.47)	90
	15HenA		May 2015	30^a^	0.46 (0.10)	1.56 (0.37)	90
	15HenB		Nov 2015	27^a^	0.78 (0.14)	1.07 (0.81)	90^b^
Durance	14SSL	N 44°14′50″ E 5°55′17″	Aug 2014	25	0.50 (0.12)	1.14 (0.46)	45
	15SSL		Sep 2015	44^b^	0.52 (0.13)	1.40 (0.40)	60^b^
Durance	14SLS	N 44°8′47″ E 5°58′9″	Aug 2014	12	0.47 (0.08)	1.66 (0.21)	45
	15SLS		Aug 2015	43^b^	0.63 (0.14)	1.61 (0.59)	60^b^
Durance	14D4	N 44°2′13″ E 5°57′56″	Jul 2014	11	0.52 (0.10)	1.14 (0.47)	60
Durance	15D5	N 44°0′1″ E 5°55′40″	Jul 2015	46	0.47 (0.15)	1.08 (0.49)	60
Buëch	14Bue	N 44°13′29″ E 5°52′27″	Sep 2014	24	0.49 (0.11)	1.15 (0.41)	45
	15Bue		Sep 2015	39	0.57 (0.14)	0.98 (0.50)	60
Verdon	15VerA	N 43°44′15″ E 6°20′58″	Jul 2015	20^a^	0.59 (0.11)	0.98 (0.51)	61
	15VerB		Jul 2015	30^a^	0.48 (0.15)	0.92 (0.48)	90
	15VerC		Sep 2015	29^a^	0.47 (0.09)	0.93 (0.44)	90
Beaume	14PltA	N 44°27′18″ E 4°16′39″	Jun 2014	35^a^	0.54 (0.08)	1.31 (0.53)	90
	14PltB		Oct 2014	6^a^	0.53 (0.14)	0.76 (0.76)	90
	15PltA		Jun 2015	49^a^	0.51 (0.12)	1.32 (0.50)	90
	15PltB		Oct 2015	30^a^	0.75 (0.14)	0.86 (0.60)	90
Loue	14PlnA	N 47°0′4″ E 5°49′36″	Jun 2014	21^a^	0.40 (0.12)	1.35 (0.38)	90
	14PlnB		Sep 2014	49^a^	0.40 (0.10)	1.43 (0.34)	90
	15PlnA		Jul 2015	41^a^	0.45 (0.14)	1.50 (0.28)	90
	15PlnB		Sep 2015	48^a^	0.75 (0.11)	1.18 (0.46)	90

*Note:* Mean and standard deviation (SD) of the Between‐Individual Component of the trophic niche (BIC) and Individual Niche Width (INW) by campaign are indicated. Diet samples size corresponds to the number of faeces retained after bioinformatic filtering of faeces metabarcoding data.

^a^ Data from Villsen, Corse, Meglécz, et al. (2022).

^b^ Data from Villsen, Corse, Archambaud‐Suard, et al. (2022).

Once captured, fish were laid in a plastic, wire mesh fishpond until biometry measurements and faeces collection were performed. Fish were weighed (precision 0.1 g), and their fork length (i.e., from the tip of the snout to the fork of the caudal fin) was measured (Lf; precision 1 mm). The abdomen of *Z. asper* individuals was gently pressed by hand to drain out faeces. Faeces were immediately placed in a 2 mL vial containing 96% ethanol and subsequently stored at −20°C. Fish were then released within the sampling area. A total of 1932 *Z. asper* individuals were caught, and 726 faeces samples were collected (maximum of one faecal sample per fish per sampling campaign). The faeces data from young‐of‐the‐year individuals were discarded from subsequent analyses due to their distinct diet compared to juveniles and adults (Villsen, Corse, Meglécz, et al. 2022). After removing young‐of‐the‐year samples, the final diet dataset included 696 faeces samples, including those obtained from Villsen, Corse, Meglécz, et al. (2022) and Villsen, Corse, Archambaud‐Suard, et al. (2022) (Table 1).

### 2.3 Diet Metabarcoding Protocol

Faecal DNA extractions and diet metabarcoding were performed as detailed in Corse et al. (2017, 2019). We then used a robust experimental design to produce relevant estimates of diet traits for characterising individual and population trophic niche variation using metabarcoding data. This robust metabarcoding protocol included (i) three distinct primer sets that target an overlapping region of the 5′ end of the Cytochrome *c* oxidase subunit I gene (COI) (see Corse et al. 2019) to minimise false negatives and to comprehensively cover the taxonomic diversity of prey and (ii) the filtering procedure described by Corse et al. (2017) and recently reimplemented in VTAM (Validation and Taxonomic Assignment of Metabarcoding data; González et al. 2023), which integrates negative controls, positive controls, and technical replicates (i.e., PCR triplicates) to minimise false positives, ensure repeatability, and validate dietary metabarcoding data within and between high‐throughput sequencing runs. VTAM notably explicitly uses the sequencing outputs of negative and positive controls (two distinct mock community samples), and of exogenous samples to set filtering thresholds for discarding false positives in faecal samples (i.e., experimental/molecular artefacts such as PCR/sequencing errors, tag switching, and cross‐sample contaminations). The sequencing output of technical (PCR) replicates was used to further ensure the reproducibility of Amplicon Sequence Variants (ASV), and chimeras and pseudogenes were also discarded. Lastly, ASVs that were identical in their overlapping regions (~130 bp) for all three primer sets were combined into contigs (further details in Corse et al. 2017, 2019).

Prey abundances in faeces were estimated using the Minimal Number of Individuals (MNI; White 1953) approach. The MNI provides a conservative estimate of prey abundance based on the number of distinct ASVs/contigs detected in each faeces sample for a given prey taxon (Corse et al. 2017). Admittedly, this estimate may be sensitive to false positives and to variation in the genetic diversity of prey taxa (the abundance of taxa that exhibit high genetic diversity may be overestimated). However, we previously demonstrated that the differences in genetic diversity between the prey of *Z. asper* only marginally biased the MNI (Villsen, Corse, Meglécz, et al. 2022). We therefore assume the MNI to be a reliable quantitative estimate of prey abundance in the faeces of *Z. asper* .

### 2.4 Individual Trophic Traits in *Z. asper*

INW and the between‐individual component of the trophic niche (BIC) trophic traits were calculated for each dietary sample. INW were calculated using the Shannon diversity index (Shannon 1948). Using the *Psicalc* function (package RinSp; Zaccarelli et al. 2013), trophic variation among individuals (BIC) was estimated using the individual specialisation index V (Bolnick et al. 2007), which corresponds to the 1‐proportional similarity index and measures the diet overlap between an individual and its population (Bolnick et al. 2002). Hence, BIC approaches 1 when an individual's diet differs greatly from that of the overall population, while BIC approaches 0 when its diet is similar to the population's diet.

### 2.5 *Zingel asper* Population Size‐Structure

To characterise the size‐structure of *Z. asper* populations, we calculated the degree of variability in fork length and weight among individuals for each sampling campaign, separately. Individual fork length and weight were first plotted using a Principal Components Analysis (PCA; function *dudi.pca*, package *ade4*; Dray and Dufour 2007). The mean of the pairwise Euclidean distances between‐individual points was then used to estimate the relative size variability for each sampling date and site combination.

### 2.6 Characterising the Biotic Dimension of Ecological Opportunity

To obtain a fine‐scale estimation of the biotic dimension of ecological opportunity in each sampling campaign, we performed fine‐scale prey community sampling. For each sampling campaign, we collected between 45 and 90 prey community and habitat samples (1708 sampling points in all; Table 1). Prey community sampling was performed 1–2 days before or after fish sampling. The sampling effort was distributed among the representative habitats (i.e., riffles, runs, glides, and rare pools) in the fishing area. Samples were collected using a Surber sampler by perpendicular transects between riverbanks, from downstream to upstream. One to five Surber samples (0.05 m^2^) were collected per transect in all accessible habitats (i.e., < 80 cm depth; < 2 m s^−1^ water velocity) (see Figure S1). Macroinvertebrates were immediately stored in 96% ethanol for subsequent identification in the laboratory. Macroinvertebrates were assigned to genus or species using morphological criteria (Tachet et al. 2010). When this was not feasible using morphology, however (e.g., due to the development stage of larvae), taxa were aggregated at higher taxonomic levels (i.e., family or subfamily). For comparison purposes, the resolution of macroinvertebrate taxonomic identification was further harmonised between morphological and faeces metabarcoding faunistic lists. The final macroinvertebrate inventory comprised 78 taxa (Table S1).

Using this dataset, we summarised the biotic dimension of ecological opportunity for each sampling campaign (i.e., site and date), separately: α‐diversity was estimated as mean prey richness, mean prey diversity (Shannon index), while β‐diversity was estimated as the mean of pairwise Bray–Curtis dissimilarities between samples. We also calculated the mean density of three key prey taxa (*Baetis*, Heptageniidae and Orthocladiinae) according to previous diet and growth studies on *Z. asper* (Monnet et al. 2022; Villsen, Corse, Meglécz, et al. 2022; Villsen, Corse, Archambaud‐Suard, et al. 2022). Prey size is known to affect predator–prey interactions via its effect on the visible detectability and nutritional quality of prey (e.g., Worischka et al. 2015). Furthermore, the abundance of various‐sized cohorts of the two main prey taxa of *Z. asper* (*Baetis* and Heptageniidae) is known to greatly vary due to phenology (i.e., voltinism), river flow and seasonal dynamics (e.g., Erba et al. 2003; Haidekker and Hering 2008). To account for the variation in abundance of different size cohorts, we separated *Baetis* and Heptageniidae into total abundance and large individuals (body length ≥ 5 mm). Prey community abundance data were *ln + 1* transformed for subsequent analyses.

Lastly, in addition to pairwise Bray–Curtis dissimilarities, we quantified the spatial heterogeneity (by sampling campaign) of each of the above‐described estimators as the coefficient of variation (*CV*):

$$\mathrm{CV}=\frac{\sigma }{\mu }*100$$

where *σ* is standard deviation (SD) and μ is the sampling campaign mean. In all, the prey community was described using 22 variables: 11 mean estimates and their corresponding coefficients of variation (Table S2). To control for variation in sampling effort, we standardised prey community variables to the minimum number of samples per sampling campaign (*n* = 45), using a permutation approach (the mean of 1000 randomised iterations).

### 2.7 Characterising the Abiotic Dimension of Ecological Opportunity

In addition to macroinvertebrate sampling, we measured habitat conditions that both drive the distribution of macroinvertebrates at the microhabitat scale and may influence the foraging behaviour of fish. For each sampling point (see above, Section 2.6) we measured: substrate size‐class richness, maximum substrate size‐class, water velocity, water depth, substrate clogging, and vegetal development. Substrate size‐classes were evaluated using a semi‐quantitative method (Malavoi and Souchon 1989), with a scale ranging from 0 (silt: 0.0039–0.0625 mm diameter) to 10 (bedrock: > 1024 mm diameter) (see Table S1 for more details). The water velocity was measured at 3 cm above the river bottom. Substrate clogging was evaluated visually using a semi‐quantitative scale ranging from 1 to 5 (see Table S4) designed to describe the level of substrate embeddedness and the clogging of the interstitial space by silt and algae (Archambaud et al. 2005). Vegetal development was characterised using a semi‐quantitative method based on a theoretical climax condition of vegetation mass. Depending on the dominant vegetation type (e.g., *Cladophora* sp. algae thalli or *Myriophyllum* sp.), a maximum development mass was approximated corresponding to 100% development. The vegetation development of samples was then estimated based on this climax condition (0%–100%) and separated into classes (class 1; 0%–25%, class 2; 25%–50%, class 3; 50%–75%, class 4; 75%–90%, class 5; 90%–100%). For each sampling campaign, habitat conditions were summarised as the mean value of each variable and their corresponding *CV* (see formula above). The abiotic dimension of ecological opportunity was described using a total of 12 estimators (Table S2).

### 2.8 Statistical Analyses

Data formatting and statistical analyses were performed using R v4.2.0 (R Core Development Team 2023).

#### 2.8.1 Prey Preferences of *Zingel asper*

To refine the characterisation of the biotic dimension of ecological opportunity, we determined the preferred prey of *Z. asper* by comparing observed prey consumption to the composition of prey taxa in the environment. We performed tests of selection for all taxa that occurred in at least 5% of *Z. asper* diet samples pooled across all sampling campaigns. Tests were performed for each sampling campaign separately using the *econullnetr* package (*generate_null_net*, sims = 1000), which calculates null model estimates of prey consumption based on observed individual diet breadth and prey availability (Vaughan et al. 2018). Three outcomes are possible for electivity tests according to the relation between observed consumption and null expectations: (i) observed > null 95 CI%; positive electivity, (ii) observed = null 95 CI%; neutral electivity, and (iii) observed < null 95 CI%; negative electivity.

#### 2.8.2 Spatiotemporal Variation in Ecological Opportunity and Diet

To summarise the underlying spatiotemporal variability in prey and habitat conditions among sampling campaigns, we performed a function PCA (package *FactoMineR*; Lê et al. 2008). All variables were scaled to a zero mean and 1 SD distribution. Results were presented in two separate biplots for prey and habitat conditions (function *fvis_pca_biplot*, package *Factoextra*; Kassambara and Mundt 2017). Spatiotemporal variation in diet was summarised using a PCA based on proportions (de Crespin de Billy et al. 2000), performed on the relative abundances (based on cumulative MNI) of prey species in the *Z. asper* diet.

#### 2.8.3 Confirmatory Path Analysis: Disentangling the Effects of Ecological Opportunity on BIC and INW

We developed a priori causal model structure to best explain variation in BIC and INW trophic traits. We considered 29 explanatory variables, which were assumed to be biologically and ecologically relevant to the foraging behaviour of *Z. asper* or its prey community (details in Table S2). These included variables related to the (i) prey community (14 variables), (ii) habitat (12 variables) and (iii) *Z. asper* population size‐structure (3 variables). As BIC is a population‐relative trophic trait while INW is individual‐centric, we quantified *Z. asper* size‐structure as population size variability (see Section 2.5) in the BIC causal model and individual weight and fork length in the INW causal model. As the distribution of benthic macroinvertebrates and thus prey availability for *Z. asper* may be in part driven by habitat conditions, we also considered a causal relationship between prey and habitat variables. Prior to causal analysis, we removed variables that were (i) highly correlated (Pearson's *R* > 0.70) and (ii) redundant according to variable inflation factor scores (function *vif*, package *car*; Fox et al. 2013). Variable inflation was calculated based on linear mixed regression models (for all response variables, separately) including site and year as random effects (function *lmer*; package *lme4*; Bates et al. 2015). Variables with high variance inflation factor scores (VIF > 5) were discarded sequentially, starting from the highest scores. During both steps of variable selection, we prioritised the retention of mean variables over CV variables.

Path analysis was performed for BIC and INW separately using d‐sep tests (function *pSEM*; package *PiecewiseSEM*; Lefcheck 2016; Shipley 2009). D‐sep tests involve constructing a global causal model from several smaller models, thus limiting the risk of over‐parametrisation. We composed causal models from linear mixed models (*lmer* function, *lme4* package; Bates et al. 2015) that included site and year as random effects to account for spatiotemporal effects. The framework used for path analysis is illustrated in Figure 1A. We considered that (i) habitat, prey, and size‐structure variables could directly influence individual trophic traits (INW and BIC), (ii) habitat variables could indirectly influence trophic traits via a prey‐mediated effect, (iii) trophic traits could not influence either habitat or prey variables (top‐down control by *Z. asper* being unlikely due to its low population density; Villsen, Corse, Meglécz, et al. 2022). In addition to the abovementioned paths, we also considered a direct effect of *Z. asper* size‐structure (related to ontogeny) on trophic traits. We considered that prey and *Z. asper* size‐structure could covary, while habitat variable covariation was considered separately. Model selection was performed as follows: (i) remove all non‐significant covariate paths, (ii) sequentially remove non‐significant causal paths to trophic traits according to *p‐*values (highest values first), (iii) remove all prey and size‐structure variables that do not have a significant path to trophic traits from the model, and (iv) sequentially remove prey–habitat non‐significant paths according to *p‐*values (highest values first).

Variable effects on trophic traits were assessed as the standardised regression estimate for each path and associated *p*‐value. The direct effect of habitat variables was estimated as the standardised regression estimate (i.e., BIC/INW~H_i_). The indirect effect was calculated as the standardised effect of habitat variable on prey variable j multiplied by its standardised effect on trophic traits (i.e., effect H_i_~P_j_ * effect P_j_~BIC/INW). The dependence and normality residuals were evaluated visually (i.e., fitted~residual values and Q‐Q plots) for the final BIC and INW linear mixed models used in path analysis (Figures S2 and S3, respectively). Goodness‐of‐fit was evaluated using Fisher's C and associated *p*‐values, with non‐significant values indicating good model fit (*p* > 0.05). Lastly, we obtained marginal (fixed effects) and conditional (fixed and random effects) *R* ^2^ values for each response variable (trophic traits and prey variables).

#### 2.8.4 Seasonal Variation in Ecological Opportunity

To better understand the mechanisms that drive seasonal trophic ITV in *Z. asper* , we quantified seasonal variation in ecological opportunity for the variables identified in causal models (see Section 2.8.3). To test the seasonal effect in key variables, we constructed linear mixed models (*lmer* function, *lme4* package; Bates et al. 2015) with a seasonal fixed effect and site and year random effects. We then tested the significance of seasonal differences via post hoc Tukey tests (function *emmeans*, package *emmeans*; Lenth 2019). Prey abundance variables were log +1 transformed prior to analysis.

## 3 Results

### 3.1 Metabarcoding Data

The raw data set was gathered from 28 distinct MiSeq runs. Once past the filtering procedure, 2724 ASVs were validated. After combining the ASVs of three COI overlapping markers, 709 contigs and 1025 ASVs were obtained. A total of 13 samples were removed after the filtering process as they did not contain any validated ASVs or contigs. None of the negative controls had validated ASVs or contigs. All ASVs expected in mock samples were retrieved. One to two extra ASVs were also validated in most of our mock samples (contig_0238, contig_0124, MFZR_000591; Table S5; see also Corse et al. 2019). A total of 640 distinct Macrometazoan ASVs/contigs (corresponding to 226 prey taxa) were obtained from the 742 *Z. asper* faeces containing validated ASVs or contigs. 69% of the prey ASVs/contigs were identified to the species level, another 17% to the genus level, 12% to the family or subfamily level, and 2% to the order or class level (Table S5).

### 3.2 Ecological Opportunity and Prey Preferences of *Z. asper*

**FIGURE 3:**
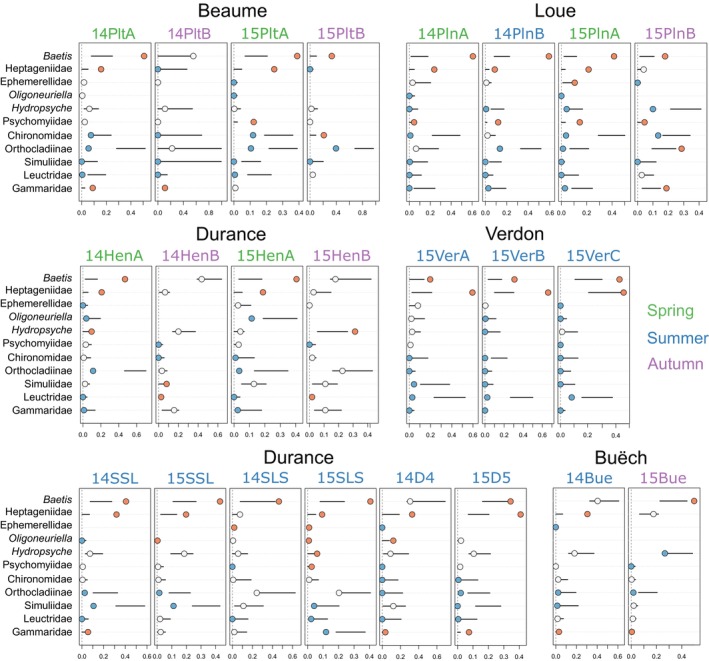
*Zingel asper* prey preferences. The position of dots along the x axis indicates observed consumption (dietary proportions; 0–1). The colour of dots indicates deviations from expected frequencies of trophic interactions; Blue, lower consumption than expected; white, as expected (in proportion to relative abundance); red, higher than expected (consumed more frequently than predicted). Horizontal lines denote 95% confidence limits of null model expectations of predation.

Our prey preference analysis reinforces the importance of *Baetis* and Heptageniidae in characterising the biotic dimension of ecological opportunity for understanding trophic ITV in *Z. asper* . *Baetis* and Heptageniidae are not only the main prey of *Z. asper* (Villsen, Corse, Meglécz, et al. 2022) but also constitute its preferred prey taxa. *Zingel asper* consistently selected its main prey *Baetis* (18/23 campaigns) and Heptageniidae (16/23 campaigns) across its entire range (Figure 3). The rare cases in which *Baetis* and Heptageniidae were neutrally or slightly negatively selected mostly occurred in autumn (e.g., 14HenB and 15HenB). Other prey were also positively selected, but preferences were either river‐dependent (e.g., Psychomiidae in the Loue River) or date‐dependent (e.g., Gammaridae and *Hydropsyche*). *Zingel asper* also frequently consumed Orthocladiinae and other chironomids, but mostly less than would be expected based on their high availability in the environment.

### 3.3 Spatiotemporal Variation in Ecological Opportunity and Diet

**FIGURE 4:**
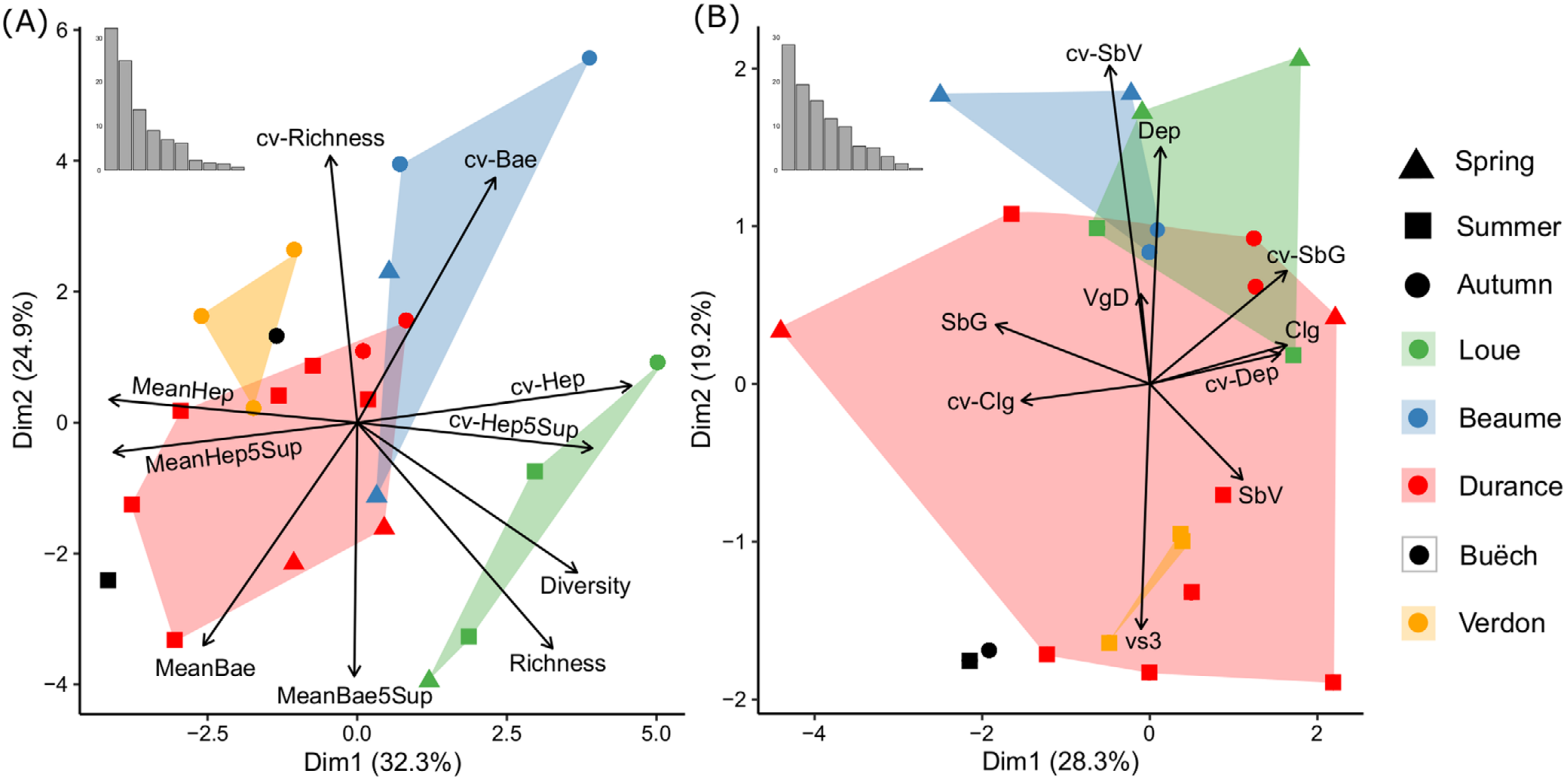
Spatiotemporal variation in (A) prey community and (B) habitat conditions according to Principal Components Analysis. Colours represent river catchments and points indicate sampling campaigns. The PCA biplots illustrate the 10 most contributing prey and habitat variables (out of 14 and 12 variables, respectively). Prey variables: Richness, mean prey richness; Diversity, mean diversity (Shannon index); MeanBae, mean *Baetis* abundance; MeanBae5sup, mean *Baetis* abundance (≥ 5 mm); MeanHep, mean Heptageniidae abundance; MeanHep5sup, mean Heptageniidae abundance (≥ 5 mm). Habitat variables: SbV, mean substrate size‐class richness; SbG, mean of the maximum substrate size‐class; Clg, mean mineral clogging class; Dep, mean of the water depth (cm); VgD, mean of the vegetal development class; vs3, mean water velocity (cm s^−1^) at 3 cm above the riverbed. When a code has the prefix cv‐ it refers to the coefficient of variation for that variable.

**FIGURE 5:**
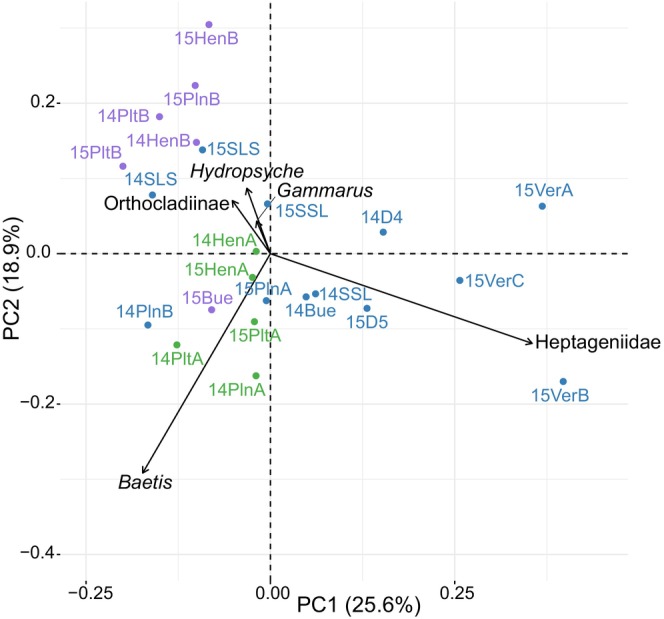
Variation in *Zingel asper* prey consumption according to Principal Coordinate Analysis based on diet proportions (pPCA). The proportions of prey taxa are based on the cumulative Minimal Number of Individuals (MNIs). Only the vectors of the five most contributive prey are illustrated. Each point represents the centroid position of each sampling campaign: Hen, SLS, SSL, D4 and D5 for the Durance River; Bue for the Buëch River; Ver for the Verdon River; Plt for the Beaume River; Pln for the Loue River. Colours correspond to seasons: Green, spring; blue, summer; purple, autumn.

Multivariate analyses revealed substantial spatial and temporal variation in both ecological opportunity (Figure 4) and in the diet of *Z. asper* (Figure 5). Regarding the biotic dimension of ecological opportunity (i.e., prey community), the horizontal axis was characterised by Heptageniidae (large and total) abundance and spatial heterogeneity, and to a lesser extent total prey richness and diversity (Figure 4A). The vertical axis mostly described variation within rivers and was characterised by spatial heterogeneity (i.e., cv‐Richness) and the availability and spatial distribution of *Baetis* (large and total). The Durance exhibited the most diverse range of prey community variables and was more comparable to the geographically close Buëch and Verdon Rivers. The Loue River exhibited the most distinct prey communities, characterised by high prey α‐diversity indices and spatially heterogeneous Heptageniidae abundances.

The spatial variation of the prey community was largely mirrored in the abiotic dimension of ecological opportunity (i.e., habitat conditions; Figure 4B). Variation in habitat conditions was mostly explained by the horizontal axis, which was characterised by a negative relationship between substrate clogging and the largest substrate size‐class. The Durance River exhibited the greatest range of habitat conditions and was more closely related to conditions observed in the Buëch and Verdon Rivers. The Beaume and the Loue Rivers were characterised by deeper and more spatially heterogeneous substrate conditions, but were overall more comparable to the Durance in terms of habitat conditions than in terms of the prey community.

The diet proportions in *Z. asper* mainly varied according to seasons, with greater preferred prey (i.e., *Baetis* and Heptageniidae) consumption in spring and summer, and greater secondary prey consumption in autumn (e.g., Orthocladiinae, *Hydropsyche*, and *Gammarus*; Figure 5). Diet composition also tended to follow a geographical pattern, with the most distinct diets observed in the Verdon River.

### 3.4 The Effect of Ecological Opportunity and Size‐Structure on BIC and INW

Using path analysis, we investigated the interaction between abiotic (i.e., habitat) and biotic (i.e., prey and size‐structure) dimensions of ecological opportunity, decoupling direct and prey‐mediated habitat effects on trophic traits (BIC and INW). Model performance was good for BIC, explaining 43% of the observed variation (*R* ^2^ marginal = 0.43, *R* ^2^ conditional = 0.53) compared to 21% for INW ($R_{m}^{2}$ = 0.21, $R_{c}^{2}$ = 0.44). Full model details are provided in Tables S6 and S7.

**FIGURE 6:**
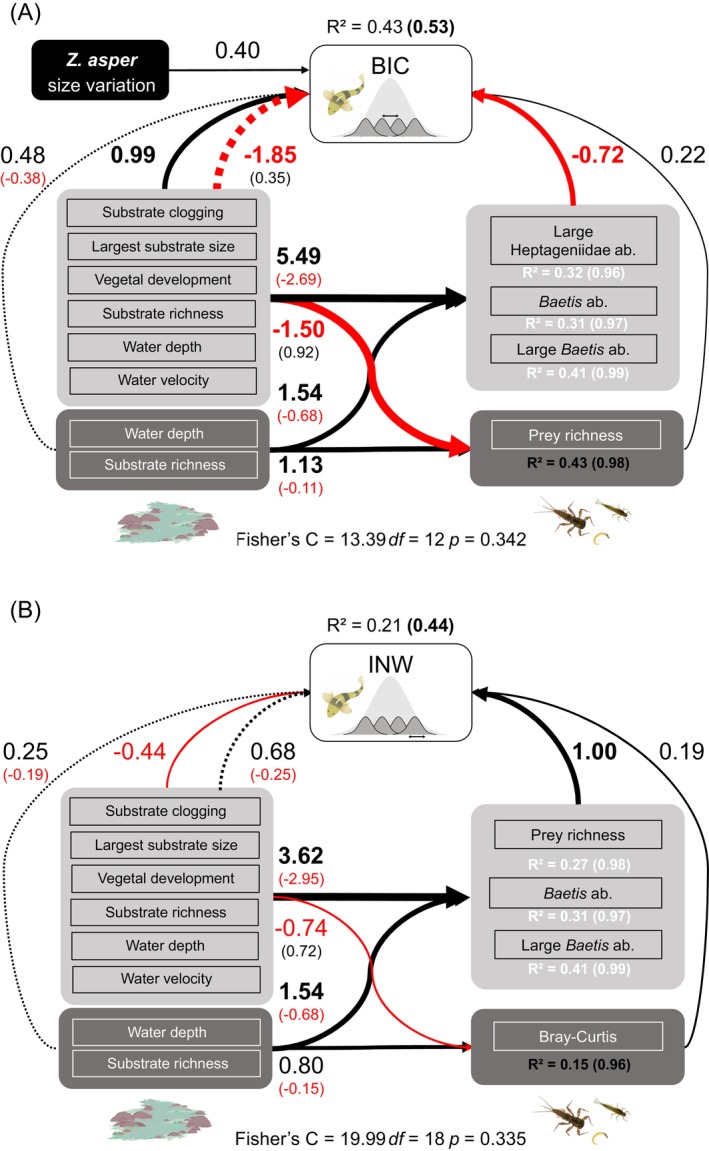
Relationships between habitat conditions, prey availability and *Z. asper* size‐structure, between‐individual trophic variation (BIC) and Individual Niche Width (INW). Light grey boxes indicate mean habitat and prey variables, while dark grey boxes indicate spatial heterogeneity related variables (*CV* or Bray‐Curtis). Only *p* < 0.05 interactions are shown. The weight of positive (black) and negative (red) cumulative standardised regression estimates between ecological compartments are indicated by thickness of the arrows. For the habitat compartement dashed lines indicate indirect effects via the prey community (calculated as effect Habitat_i_ ~ Prey_j_ * effect Prey_j_ ~ BIC/INW). Marginal and conditional (in brackets) *R* ^2^ values are indicated for INW, BIC and prey response variables. For full model details see Tables S6 and S7.

The BIC model was characterised by direct habitat effects that were exclusively positive, a positive *Z. asper* population size variation effect, and both positive and negative prey effects (Figure 6A). Prey effects were divided into: (i) negative effects associated with the abundance of large, preferred prey taxa (*Baetis* and Heptageniidae mayflies) and total *Baetis* abundance, and (ii) a positive effect of the spatial heterogeneity of prey richness. A direct positive effect of habitat on BIC was associated with substrate conditions (largest substrate size and sediment clogging) and vegetal development. While the strongest effect was the indirect (i.e., prey‐mediated) habitat effect, it is noteworthy that the magnitude of direct habitat effects on BIC was comparable to that of direct prey effects on BIC (Figure 6A). Mean habitat variables were largely positively associated with preferred prey abundance and negatively associated with spatial heterogeneity in prey richness, thus resulting in a negative effect on BIC overall. Greater spatial heterogeneity in substrate diversity was associated with higher heterogeneity in prey richness and was thus indirectly associated with higher BIC. Random effects indicated significant spatial variation in BIC. Notably, BIC tended to be higher in the Verdon (Ver) and one of the Durance sites (SLS) and marginally lower in the Beaume site (Plt; see Figure S4).

In contrast, the INW model was characterised by a consistent positive relationship with prey variables, a weaker negative habitat effect, and no significant *Z. asper* size‐related effect (Figure 6B). Spatial heterogeneity (mean pairwise Bray–Curtis) and richness of the prey community, along with preferred prey abundance (total and large *Baetis*), were all positively correlated with INW. While water velocity and vegetal development were both negatively correlated with INW. As for the BIC model, the indirect habitat effect was characterised by a largely positive relationship between habitat and prey variables, resulting in a positive overall indirect effect on INW. For example, vegetal development was positively associated with preferred prey abundance (total and large *Baetis* abundance) and total diversity. Lastly, random effects indicated significant annual variation in INW, with lower values observed in 2014 compared to 2015 (Figure S5).

### 3.5 Seasonal Variation in Ecological Opportunity

**FIGURE 7:**
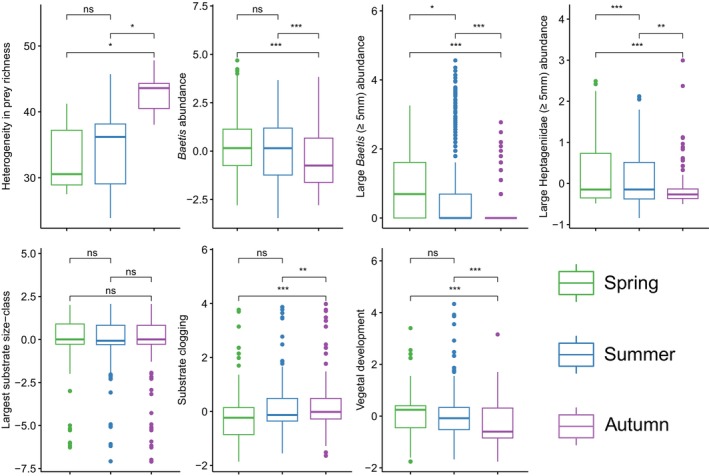
Seasonal variation in estimates of ecological opportunity. Only variables that had a significant effect on between‐individual niche variation in path analyses are included. Boxplots represent residuals extracted from the following linear model: lm(y ~ Site + Year). Significant differences between seasons were determined by Tukey post hoc *emmeans* tests on linear mixed models *y* ~ *season* + (1| *site*) + (1| *year*). The significance of pairwise comparisons is indicated as ***p < 0.001, **p < 0.01, *p < 0.05, ns: not significant.

Most of the biotic and abiotic variables of ecological opportunity associated with ITV in the path analyses follow a clear seasonal trajectory from spring to autumn (Figure 7). Preferred prey abundance tended to decline progressively from spring to autumn. This seasonal trajectory was most notable for large *Baetis* and Heptageniidae individuals, which were significantly less abundant in autumn than in summer or spring. Similarly, total *Baetis* abundance was significantly lower in autumn than in summer and spring. Spatial heterogeneity in prey richness tended to increase from spring to autumn, though only the autumn‐spring and autumn‐summer comparisons were significant. For habitat variables, both substrate clogging and vegetal development followed a clear seasonal trajectory: clogging was higher in autumn than in spring or summer, and vegetal development declined in autumn compared to spring and summer.

## 4 Discussion

While biotic (e.g., Sánchez‐Hernández et al. 2017, 2021), abiotic (e.g., Musseau et al. 2015), and spatial heterogeneity (e.g., Walker et al. 2023) have individually proven key drivers of trophic ITV, few studies have sought to understand their joint and interactive effects on trophic ITV. By coupling fine‐scale metabarcoding, habitat, and prey sampling, we were able to show that *Z. asper* both modulates its trophic niche according to variation in its prey community (e.g., preferred prey abundance and spatial structure) and habitat structure (e.g., substrate clogging and granulometry). Our findings provide useful insights into the specific processes that drive and constrain the trophic niche of benthic fishes like *Z. asper* , while also shedding light on a more generalisable framework of trophic ITV determinism.

### 4.1 Trophic ITV is Jointly Explained by Biotic and Abiotic Dimensions of Ecological Opportunity

The role of abiotic conditions in trophic ITV determinism has traditionally been conceptualised as resulting from prey‐mediated processes (e.g., Layman et al. 2007). In this context, abiotic conditions are expected to drive variation in prey availability (e.g., due to prey habitat preferences), and thus indirectly alter foraging behaviour or trophic ITV (Araújo et al. 2011; Dermond et al. 2018; Layman et al. 2007; Lunghi et al. 2020; MacArthur 1965). While our findings also support the importance of prey‐mediated processes, we additionally highlighted a direct habitat effect which appeared to be both distinct and equally important as prey‐mediated processes. The fact that the direct habitat effect on trophic traits was consistently antagonistic to prey community effects (i.e., prey‐mediated and direct prey effects) indicates that habitat conditions provide unique and complementary information to biotic conditions in trophic ITV determinism. We therefore validated our initial hypothesis demonstrating the joint and interactive effects of abiotic and biotic dimensions of ecological opportunity on trophic ITV.

The abiotic and biotic drivers of trophic ITV in *Z. asper* can be conceptualised in terms of how they modify the net energetic benefits of foraging (c.f., Optimal Foraging Theory; OFT; Perry and Pianka 1997), indicating that our results may be generalisable across other taxa. The OFT posits that the net energetic benefits of a given prey are modulated by the opportunity cost of stopping to consume it, versus the price of moving on in search of more energetically beneficial prey (Perry and Pianka 1997). We interpreted dietary divergence among individuals (i.e., BIC) as an indicator of the balance between selective (i.e., when individuals specialise on preferred prey) and opportunistic (i.e., when a broad range of secondary prey are consumed) foraging in *Z. asper* . In accordance with OFT predictions and previous empirical studies, selective foraging (i.e., lower BIC) was most prevalent when prey richness was high (e.g., Sánchez‐Hernández et al. 2017, 2021) and when high‐quality prey were abundant (e.g., Tinker et al. 2008). The abiotic drivers of trophic ITV can similarly be conceptualised in terms of their effect on the net energetic benefits of foraging. For example, the net energetic benefits of prey are not only dependent on prey traits (e.g., abundance, size, nutritional content), but also on environmental constraints that influence foraging success (Perry and Pianka 1997; Townsend and Winfield 1985). In *Z. asper* , the direct effect of the abiotic factors on BIC was mainly linked to landscape elements that are also known to alter foraging success (vegetation and substrate size) or individual movement (substrate clogging) in fish (Angermeier 1985; Beekey et al. 2004; Champion et al. 2018; Nguyen and Crocker 2006). The notion that abiotic conditions regulate foraging selectivity is further supported by experimental evidence (e.g., vegetation; Harrel and Dibble 2001) as well as their negative effect on INW in the present study (which we interpret as an indicator of foraging success). Our results therefore strongly support the notion that abiotic factors also contribute to modifying the net energetic benefits of prey and, therefore, trophic ITV.

Variation in abiotic and biotic elements of ecological opportunity can therefore provide a mechanistic explanation for seasonal trophic ITV in *Z. asper* : wherein beneficial foraging conditions (i.e., high ecological opportunity) in spring and summer allow individuals to specialise in preferred prey, and therefore maximise energetic gain. Then, when ecological opportunity is low in autumn, individuals tend to shift to a broader range of less energetically beneficial prey as the opportunity cost of continuing the search for more beneficial prey increases.

### 4.2 Spatial Heterogeneity in Ecological Opportunity Drives Trophic ITV

The spatial distribution of ecological opportunity features is a key component of OFT theory, driving the opportunity cost of prey consumption (Perry and Pianka 1997). Despite strong support for the role of spatial heterogeneity factors, few studies of trophic ITV have explicitly tested the hypothesis that spatial heterogeneity in abiotic and biotic ecological opportunity drives trophic ITV (but see: Walker et al. 2023). Our findings support the key role of spatial heterogeneity in the biotic dimension of ecological opportunity (i.e., either direct prey or prey‐mediated habitat effects), but fail to detect a significant direct habitat effect. We therefore only partially validate our initial hypothesis regarding spatial heterogeneity drivers of trophic ITV.

We expect that the effect of resource distribution is especially important in heterogeneous ecosystems like rivers, wherein habitat types (e.g., runs, riffles, pools) support distinct prey communities (Perez Rocha et al. 2018). For foragers with small ranges and limited mobility, local prey availability may therefore shape dietary differences and may lead to individualised foraging strategies based on local trade‐offs between prey quality and foraging costs (MacArthur and Pianka 1966; Stephens and Krebs 1986). This hypothesis corresponds with what is known of the ecology of *Z. asper* , wherein individuals tend to space themselves out within populations rather than congregate in preferred habitats (Labonne and Gaudin 2005). Trophic ITV in *Z. asper* therefore appears to mirror the spatial distribution of its biotic ecological opportunity, wherein local‐scale conditions exert a bottom‐up effect on individual diet—leading to either dietary divergence or convergence at the population level. In support of this hypothesis, we also observed higher within‐individual diet variation (i.e., INW) when prey conditions were spatially heterogeneous. This suggests that when prey are heterogeneously distributed, individuals tend to diversify their individual diet to meet energetic demands as the opportunity cost of rejecting secondary prey increases.

Previous studies have demonstrated that habitat structural complexity can regulate overlap in individual home ranges (i.e., higher complexity reduces habitat overlap), thus driving between‐individual diet variation (de Camargo et al. 2019). We therefore initially hypothesised that the spatial distribution of abiotic factors related to habitat structure would directly influence trophic traits in *Z. asper* . However, rather than structural complexity factors, the factors that regulate habitat use in *Z. asper* may relate to conditions that inhibit movement among habitat patches. For example, substrate clogging is actively avoided by certain benthic fishes (Kawanishi et al. 2015; Champion et al. 2018) and may therefore represent a micro‐scale barrier to movement for *Z. asper* . In support of this hypothesis, we found a positive effect between substrate clogging and dietary divergence (i.e., BIC). However, further studies will be needed to properly characterise the mechanisms that drive habitat use in benthic fishes like *Z. asper* or in other more diverse taxa. To this end, Costa‐Pereira and Shaner (2025) present a promising approach coupling individual trophic and habitat niches to improve our understanding of how both niches actively interact with each other as well as ecological opportunity factors.

### 4.3 Limitations

In the present study, we demonstrated the importance of (i) the prey community, (ii) habitat conditions and (iii) the spatial distribution of ecological opportunity as key determinants of trophic ITV. However, other biotic factors are also recognised as important drivers of trophic ITV, such as intra‐ and interspecific competition and predation risk (Costa‐Pereira, Toscano, et al. 2019; Evangelista et al. 2014; Musseau et al. 2015). Admittedly, incorporating these dimensions may further improve our understanding of trophic ITV in *Z. asper* , although past research indicates their effect may be limited. For instance, in terms of interspecific competition, we previously found extensive dietary overlap between *Z. asper* and the ecologically similar species *Cottus gobio* . However, we also highlighted that when large preferred prey (e.g., *Baetis*) are scarce, *Z. asper* and *C. gobio* partition their diets and therefore avoid competition (Villsen, Corse, Archambaud‐Suard, et al. 2022). We additionally expect intra‐specific competition to have a limited effect on the trophic ITV of *Z. asper* due to its naturally low population density (Villsen, Corse, Meglécz, et al. 2022).

### 4.4 Conservation and Management Perspectives

This study offers several important conservation and management perspectives for *Z. asper* . First, we found that *Baetis* and Heptageniidae were strongly selected by *Z. asper* . As the availability of high‐quality prey can directly affect fitness and population resilience (Bagenal 1978; Elliott and Hurley 2000; Garvey and Whiles 2016), habitat quality assessments for *Z. asper* (e.g., for river management, or the selection of suitable reintroduction sites) should therefore consider both the availability and spatial heterogeneity of *Baetis* and Heptageniidae. Secondly, our findings highlighted a potential negative effect of substrate clogging on *Z. asper* and its prey, which was strongly related to heterogeneously distributed prey communities, lower preferred prey abundance, and a dietary shift toward less profitable prey. River restoration efforts targeting substrate granulometry and clogging have proven highly effective in supporting the recovery of riverine benthic fish populations (e.g., Gray et al. 2024). For example, tailored flushing flows in regulated rivers may help mitigate the negative impacts of substrate clogging on fish like *Z. asper* and their prey (Loire et al. 2019). This issue is far from trivial, as substrate clogging remains a persistent problem affecting both macroinvertebrates and fish populations in the Durance River (Cazaubon and Giudicelli 1999; Corse, Pech, et al. 2015) and potentially threatens the largest and most genetically diverse *Z. asper* population. Lastly, our findings serve as a demonstration of the high trophic plasticity of *Z. asper* . In a previous study (Villsen, Corse, Meglécz, et al. 2022), we demonstrated that *Z. asper* is able to maintain its body condition (a fitness proxy; Kotiaho 1999) across seasons, independent of seasonal trophic ITV. Coupled with the results of the present study, *Z. asper* appears to be capable of shifting between opportunistic and selective foraging in order to maintain its fitness in response to changes in ecological opportunity (Roughgarden 1972; Trappes et al. 2022).

## 5 Conclusion

To our knowledge, this study represents the first mechanistic attempt to jointly examine the effects of both biotic and abiotic dimensions of ecological opportunity on trophic ITV. From a methodological point of view, this study serves as a proof of concept for the use of metabarcoding diet data for mechanistic trophic analyses, demonstrating how trophic traits calculated from short‐term metabarcoding data can be directly linked to fine‐scale snapshot estimates of ecological opportunity. Our main results include the decoupling of direct and prey‐mediated habitat effects on trophic ITV and highlight the importance of the spatial heterogeneity of prey conditions as a complementary determinant of ITV. The strong habitat effect revealed in this study underscores the importance of accounting for habitat and landscape in studies of trophic ecology, not only in terms of their prey‐mediated effect, but also their direct effect on trophic ITV (Costa‐Pereira and Shaner 2025). This study therefore marks a substantial step forward in our understanding of how biotic, abiotic, spatial and temporal dimensions of ecological opportunity interact to jointly drive within‐population variation of resource use. In the present study, we focused on physical habitat characteristics, but other extrinsic (e.g., temperature driving thermal mismatch between predators and prey, Pintanel et al. 2021) and intrinsic (e.g., individual nutritional condition, Walker et al. 2023) factors may be equally important. The results of this study serve as a demonstration of the complex nature of trophic ITV determinism and therefore the need for a holistic approach in future studies.

## Author Contributions

V.D., E.C. and G.A.‐S. conceived and designed the study. V.D., E.C., G.A.‐S. and R.C. performed the fieldwork. E.C. did the molecular work. E.M. did the bioinformatics. G.A.‐S., J.‐P.B. and M.B. morphologically identified and measured the macroinvertebrates. S.B. contributed key analytical methodologies. K.V. performed statistical analyses, with contributions from E.C. K.V., V.D. and E.C. wrote the original draft, and all authors contributed to further writing and editing.

## Conflicts of Interest

The authors declare no conflicts of interest.

## Supporting information


**Figure S1:** mec70115‐sup‐0001‐Figures.pdf.

**Table S1:** mec70115‐sup‐0002‐Tables.zip.

## Data Availability

Supporting Information deposited in Dryad (https://doi.org/10.5061/dryad.2ck7120) including: (i) unfiltered HTS data; (ii) the sample/tag combination correspondences; (iii) the filtered and validated sequence data, and the taxonomic assignment of prey detected in *Z. asper* 's faeces; (iv) the final diet dataset used to estimate individual trophic traits; and (v) the macroinvertebrate and habitat datasets used for statistical analysis and modelling.
