# Supplementary material for: Disentangling the Effects of Biotic and Abiotic Dimensions of Ecological Opportunity on Individual Trophic Trait Variation
Source: Mol Ecol. 2025 Sep 24;34(20):e70115. doi: 10.1111/mec.70115 (PMC12530292; doi:10.1111/mec.70115)
Supplement: Supplementary file 1 — Figure S1: mec70115‐sup‐0001‐Figures.pdf. [file MEC-34-e70115-s001.pdf]

Disentangling the effects of biotic and abiotic dimensions of ecological opportunity on individual trophic trait variation

Kurt Villsen[1,2], Gaït Archambaud-Suard[2], Emese Meglécz[1], Simon Blanchet[3], Jean-Pierre Balmain[2], Mathilde Bertrand[1,2], Rémi Chappaz[2], Vincent Dubut[1,4], Emmanuel Corse[1,5,6]

[1]Aix Marseille Univ, Avignon Université, CNRS, IRD, IMBE, Marseille, France
[2]INRAE, Aix Marseille Univ, RECOVER, Aix-en-Provence, France
[3]CNRS, Station d'Écologie Théorique et Expérimentale (UAR 2029–SETE), Moulis, France
[4]ADENEKO, Saint-Girons, France
[5]Université de Mayotte, Dembeni, Mayotte, France
[6]MARBEC, CNRS, Ifremer, IRD, Université de Montpellier, Montpellier, France

Supporting Information

Supporting Figure S1

Supporting Figure S2

Supporting Figure S3

Supporting Figure S4

Supporting Figure S5

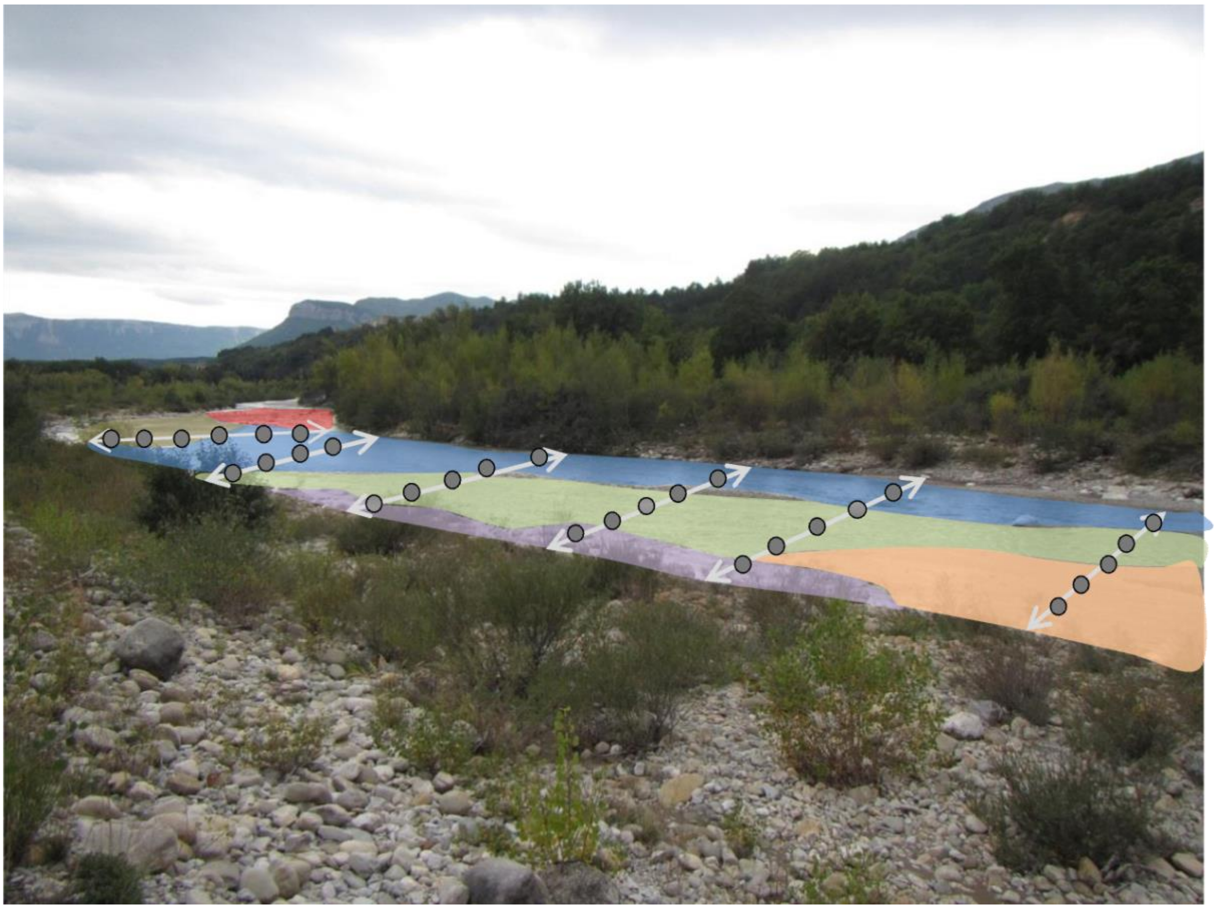

Figure S1. Macroinvertebrate sampling methodology. One to five surber samples were taken per transect with the intention of sampling all representative habitat types (represented by colouration in the figure) in each respective transect

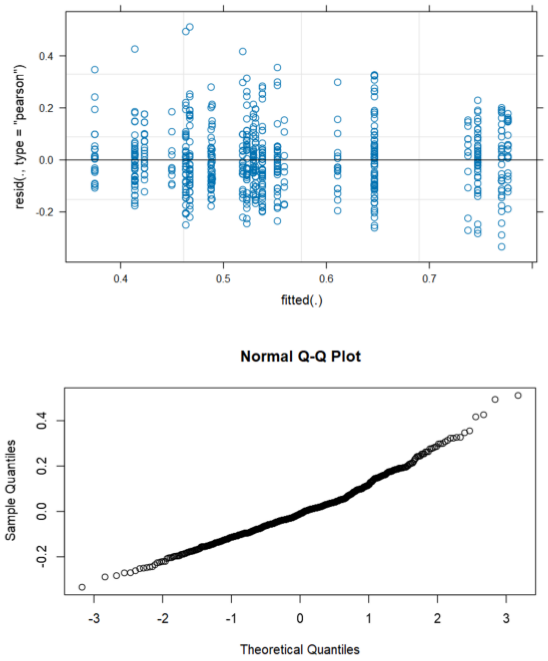

Figure S2. Visual assessments of model fit for the final BIC linear mixed model from causal analysis. Residuals versus fitted model values (above) and Q-Q plot (below).

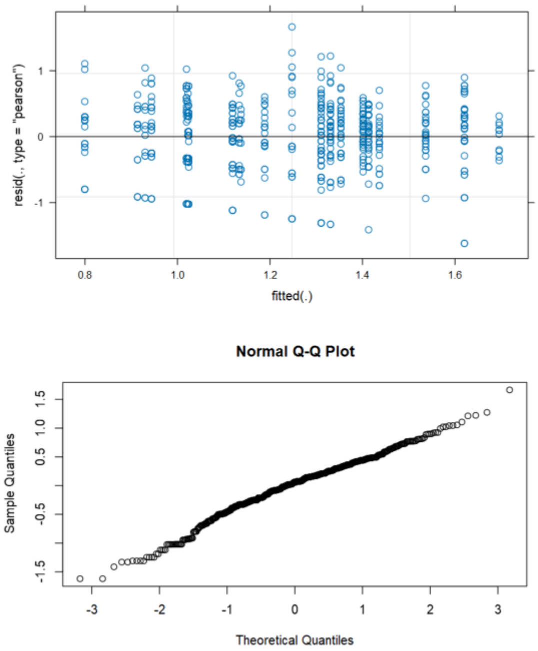

Figure S3. Visual assessments of model fit for the final INW linear mixed model from causal analysis. Residuals versus fitted model values (above) and Q-Q plot (below).

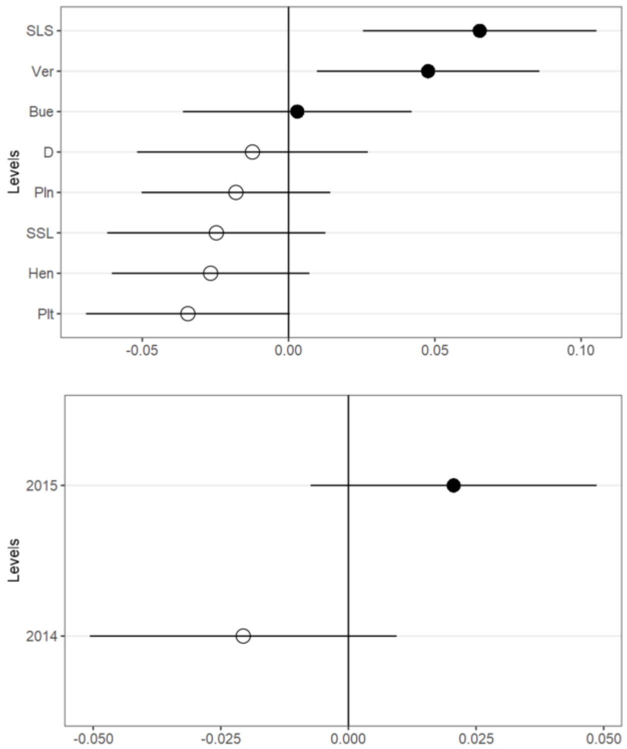

Figure S4. Illustration of standardised random effects (site: above & year: below) for the final Between Individual Component of the trophic niche (BIC) casual model. Filled points indicate overall positive effects while empty points indicate a negative effect, lines indicate the 95% confidence interval. Durance River sites (Hen, SSL, SLS and D), Verdon River (Ver), Buëch River (Bue), Beaume River (Plt) and Loue River (Pln).

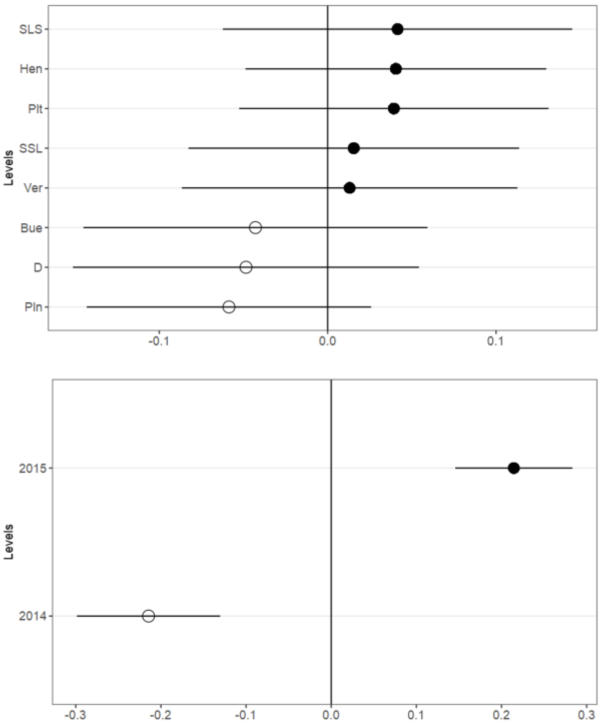

Figure S5. Illustration of standardised random effects (site: above & year: below) for the final Individual Niche Width (INW) casual model. Filled points indicate overall positive effects while empty points indicate a negative effect, lines indicate the 95% confidence interval. Durance River sites (Hen, SSL, SLS and D), Verdon River (Ver), Buech River (Bue), Beaume River (Plt) and Loue River (Pln).
