# Supplementary material for: Disentangling the Effects of Biotic and Abiotic Dimensions of Ecological Opportunity on Individual Trophic Trait Variation
Source: Mol Ecol. 2025 Sep 24;34(20):e70115. doi: 10.1111/mec.70115 (PMC12530292; doi:10.1111/mec.70115)
Supplement: Supplementary file 2 — Table S1: mec70115‐sup‐0002‐Tables.zip. [file MEC-34-e70115-s002.zip › mec70115-sup-0002-TablesS1-S4.pdf]

Disentangling the effects of biotic and abiotic dimensions of ecological opportunity on individual trophic trait variation

Kurt Villsen[1,2], Gaït Archambaud-Suard[2], Emese Meglécz[1], Simon Blanchet[3], Jean-Pierre Balmain[2], Mathilde Bertrand[1,2], Rémi Chappaz[2], Vincent Dubut[1,4], Emmanuel Corse[1,5,6]

[1]Aix Marseille Univ, Avignon Université, CNRS, IRD, IMBE, Marseille, France
[2]INRAE, Aix Marseille Univ, RECOVER, Aix-en-Provence, France
[3]CNRS, Station d'Écologie Théorique et Expérimentale (UAR 2029–SETE), Moulis, France
[4]ADENEKO, Saint-Girons, France
[5]Université de Mayotte, Dembeni, Mayotte, France
[6]MARBEC, CNRS, Ifremer, IRD, Université de Montpellier, Montpellier, France

Supporting Information

Supporting Table S1

Supporting Table S2

Supporting Table S3

Supporting Table S4

Table S1. Final faunistic list for the macroinvertebrate community

Identified Taxa	Higher taxonomic grouping
Acentrella sinaica	Ephemeroptera
Alainites muticus	Ephemeroptera
Baetis	Ephemeroptera
Centroptilum	Ephemeroptera
Cloeon	Ephemeroptera
Procloeon	Ephemeroptera
Other Baetidae	Ephemeroptera
Caenis	Ephemeroptera
Ephemerellidae	Ephemeroptera
Ephemera	Ephemeroptera
Epeorus	Ephemeroptera
Heptageniidae	Ephemeroptera
Rhithrogena	Ephemeroptera
Leptophlebiidae	Ephemeroptera
Oligoneuriella	Ephemeroptera
Polymitarcyidae	Ephemeroptera
Potamanthus	Ephemeroptera
Other Ephemeroptera	Ephemeroptera
Capniidae	Plecopetera
Chloroperlidae	Plecopetera
Leuctridae	Plecopetera
Nemouridae	Plecopetera
Dinocras	Plecopetera
Perla	Plecopetera
Perlodidae	Plecopetera
Taeniopterygidae	Plecopetera
Brachycentridae	Trichoptera
Cheumatopsyche	Trichoptera
Hydropsyche	Trichoptera
Hydroptilidae	Trichoptera
Leptoceridae	Trichoptera
Limnephilidae	Trichoptera
Wormaldia	Trichoptera
Other Philopotamidae	Trichoptera
Polycentropodidae	Trichoptera
Psychomyiidae	Trichoptera
Rhyacophilidae	Trichoptera
Other Trichoptera	Trichoptera

Table S1. (continued)

Identified Taxa	Higher taxonomic grouping
Athericidae	Diptera
Blephariceridae	Diptera
Ceratopogonidae	Diptera
Other Chironomidae	Diptera
Orthocladiinae	Diptera
Empididae	Diptera
Limoniidae	Diptera
Simuliidae	Diptera
Tabanidae	Diptera
Tipulidae	Diptera
Other Diptera	Diptera
Dryopidae	Coleoptera
Dytiscidae	Coleoptera
Elmis	Coleoptera
Esolus	Coleoptera
Limnius	Coleoptera
Oulimnius	Coleoptera
Riolus	Coleoptera
Stenelmis	Coleoptera
Gyrinidae	Coleoptera
Other Coleoptera	Coleoptera
Odonates	Odonates
Megaloptera	Megaloptera
Heteroptera	Heteroptera
Gammaridae	Crustacea
Other Crustacea	Crustacea
Hydracarina	Chelicerata
Hirudinea	Annelida
Lumbricidae	Annelida
Naididae	Annelida
Other Oligochaeta	Annelida
Ancylidae	Mollusca
Hydrobiidae	Mollusca
Neritidae	Mollusca
Sphaeriidae	Mollusca
Other Mollusca	Mollusca
Planariidae	Platyhelminthes
Nematoda	Nematoda
Nemertea	Nemertea
Cnidaria	Cnidaria

Table S2. Details and assumptions for prey (n=14), habitat (n=12) and Zingel asper size-structure (n=3) variables included in causal analysis for BIC and INW trophic traits (CV indicates the coefficient of variation).

Variable type	Code	Variable	Assumptions	References
**Prey community α-diversity**	Richness	Mean prey richness	Individual and population-level niche expansion is expected when the prey community is diverse. Calculated based on the total range of potential prey taxa (78 taxa ; Table S1).	Araujo et al. 2011; Evangelista et al. 2014; Sjödin et al. 2018
	Diversity	Mean prey diversity (Shannon)		
**Prey community β-diversity (spatial hetrogeneity)**	cv-Richness	CV of prey richness	The spatial distribution of prey determines detection rate and thus opportunity cost for predators	MacArthur & Pianka, 1966
	cv-Diversity	CV of prey diversity (Shannon)		
	BrayC	Mean of Bray-Curtis pairwise distances		
	cv-BrayC	CV of Bray-Curtis pairwise distances		
**Preferred prey abundance**	MeanBae	Mean *Baetis* abundance	When preferred prey are abundant individual diets are expected to converge towards optimal prey choices. See prey selection results (Figure 3).	Villsen et al. 2022; Tinker et al. 2008
	MeanHep	Mean Heptageniidae abundance		
**Spatial heterogeneity of preferred prey abundance**	cv-Bae	CV of *Baetis* abundance	The spatial distribution of prey determines detection rate and thus opportunity cost for predators	MacArthur & Pianka, 1966
	cv-Hep	Mean Heptageniidae abundance		
**Large preferred prey abundance**	MeanBae5Sup	Mean *Baetis* (≥ 5mm) abundance	Prey size is an important component of prey selection, influencing the nutritional value of prey, ease of detection and handling time. See prey selection results (Figure 3).	Gaeta et al. 2018; Dodrill et al. 2021
	MeanHep5Sup	Mean Heptageniidae (≥ 5mm) abundance		
**Spatial heterogeneity of large preferred prey abundance**	cv-Bae5Sup	CV of *Baetis* (≥ 5mm) abundance	The spatial distribution of prey determines detection rate and thus opportunity cost for predators	MacArthur & Pianka, 1966
	cv-Hep5Sup	CV of Heptageniidae (≥ 5mm) abundance		

Table S2. (continued)

Variable type	Code	Variable	Assumptions	References
**Substrate composition**	SbV	The mean number of substrate size-classes (see Table S1)	Substrate type and distribution affects foraging sucess in visual benthic predators	Angermeier, 1985
	SbG	The mean largest substrate size-class (see Table S1)		
**Spatial heterogeneity of substrate size-classes**	cv-SbV	CV of the number of substrate size-classes (see Table S1)		
	cv-SbG	CV of the largest substrate size-class (see Table S1)		
**Substrate clogging**	Clg	Mean clogging class(see Table S2)	Substrate clogging is actively avoided by some benthic fishes. It may therefore promote spatial isolation among individuals and thus promote BIC	Kawanishi et al. 2015; de Camargo et al. 2019
**Spatial heterogeneity of substrate clogging**	cv-Clg	CV of the clogging class (see Table S2)		
**Water velocity**	Vs3	Mean water velocity (m.s−1) measured at 3cm from the riverbed	Water velocity modifies the energetic cost of foraging and microhabitat use	Facey & Grossman, 1992; Wood et al. 2013
**Spatial heterogeneity of water velocity**	cv-Vs3	CV of water velocity (m.s−1) measured at 3cm from the riverbed		
**River depth**	Dep	Mean Depth (cm)	River depth drives the distribution of macroinvertebrates	Nautiyal & Mishra, 2022
**Spatial heterogeneity of river depth**	cv-Dep	CV of Depth (cm)		
**Vegetal development**	VgD	Mean vegetal development	Vegetation cover and type affect foraging sucess in visual benthic predators	Harrel & Dibble, 2001
**Spatial heterogeneity of vegetal development**	cv-VgD	CV of vegetal development		
**Size variability**	SizeVar	Intra-population variability in fork-length and weight among *Zingel asper* individuals	Ontogenic dietary variation is a major source of trophic niche variation in fishes	Sanchez-Hernandez et al. 2019
**Fork length**	Length	*Zingel asper* individual fork-length (mm)	Larger individuals tend to exhibit broader trophic niches	
**Weight**	Weight	*Zingel asper* individual weight (g)		

Table S3. Substrate size-classes based on Malavoi & Souchon (1989)

Substrate size-class	Description	Diameter (mm)
10	Very large boulders - bedrock	> 1024
9	Large - medium boulders	[256 - 1024]
8	Small boulders	[128 - 256[
7	Very small boulders	[64 - 128[
6	Very coarse gravel	[32 - 64[
5	Coarse gravel	[16 - 32[
4	Medium gravel	[8 - 16[
3	Fine gravel	[2 - 8[
2	Coarse - medium sand	[0.5 - 2[
1	Fine - very fine sand	[0.0625 - 0.5[
0	Silt	[0.0039 - 0.0625[

Table S4. Description of substrate clogging based on Archambault et al. 2005

Class	Clogging (%)	Representation of the degree of substrate clogging
1	[0 - 25%[	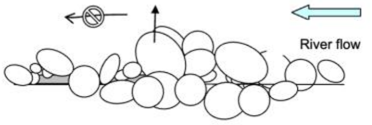 The substrate elements are resting on the riverbed. A fine layer of silt may be observed (see left) or no silt at all (see right)
2	[25 - 50%[	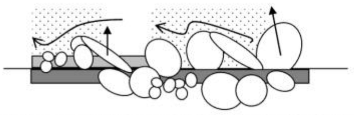 The substrate elements are stuck together by an embedded layer of silt. When the substrate is disturbed, the cloud of silt produced is not dense.
3	[50 - 75%[	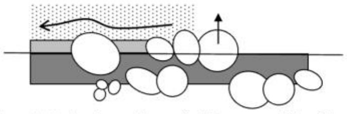 The substrate elements are lightly encased by silt. When the substrate is disturbed, a medium density cloud of silt is produced
4	[75 - 90%[	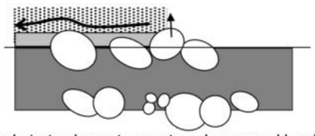 The substrate elements are strongly encased by silt. When the substrate is disturbed, a high density cloud of silt is produced
5	[90 - 100%]	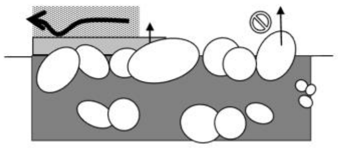 The substrate elements are strongly encased in silt. When the substrate is disturbed, a very high density cloud of silt is produced (see left). Or the elements are completely cemented into the underlayer and are impossible to lift (see right)

References cited

Angermeier, P. L. (1985). Spatio-temporal patterns of foraging success for fishes in an Illinois stream. *The American Midland Naturalist*, *114*(2), 342–359. https://doi.org/10.2307/2425609

Araújo, M. S., Bolnick, D. I., & Layman, C. A. (2011). The ecological causes of individual specialisation. *Ecology Letters*, *14*(9), 948–958. https://doi.org/10.1111/j.1461-0248.2011.01662.x

Archambaud, G., Giordano, L., & Dumont, B. (2005). *Description du substrat minéral et du colmatage - Note Technique.* Cemagref Aix-en-Provence, UR Hydrobiologie.

de Camargo, N. F., de Oliveira, H. F., Ribeiro, J. F., de Camargo, A. J., & Vieira, E. M. (2019). Availability of food resources and habitat structure shape the individual-resource network of a neotropical marsupial. *Ecology and Evolution*, *9*(7), 3946–3957. https://doi.org/10.1002/ece3.5024

Dodrill, M. J., Yackulic, C. B., Kennedy, T. A., Yard, M. D., & Korman, J. (2021). As the prey thickens: Rainbow trout select prey based upon width not length. *Canadian Journal of Fisheries and Aquatic Sciences*, 78(7), 809-819.

Evangelista, C., Boiche, A., Lecerf, A., & Cucherousset, J. (2014). Ecological opportunities and intraspecific competition alter trophic niche specialization in an opportunistic stream predator. *Journal of Animal Ecology*, *83*(5), 1025–1034. https://doi.org/10.1111/1365-2656.12208

Facey, D. E., & Grossman, G. D. (1992). The relationship between water velocity, energetic costs, and microhabitat use in four North American stream fishes. *Hydrobiologia*, *239*(1), 1–6. https://doi.org/10.1007/BF00027524

Gaeta, J. W., Ahrenstorff, T. D., Diana, J. S., Fetzer, W. W., Jones, T. S., Lawson, Z. J., ... & Vander Zanden, M. J. (2018). Go big or… don't? A field-based diet evaluation of freshwater piscivore and prey fish size relationships. *PloS ONE*, 13(3), e0194092.

Harrel, S. L., & Dibble, E. D. (2001). Foraging efficiency of juvenile bluegill, *Lepomis macrochirus*, among different vegetated habitats. *Environmental Biology of Fishes*, *62*, 441–453. https://doi.org/10.1023/A:1012259922727

Kawanishi, R., Dohi, R., Fujii, A., & Inoue, M. (2015). Effects of sedimentation on an endangered benthic fish, *Cobitis shikokuensis*: Is sediment-free habitat a requirement or a preference? *Ecology of Freshwater Fish*, *24*(4), 584–590. https://doi.org/10.1111/eff.12171

MacArthur, R. H., & Pianka, E. R. (1966). On optimal use of a patchy environment. *The American Naturalist*, *100*(916), 603–609. https://doi.org/10.1086/282454

Malavoi, J.-R., & Souchon, Y. (1989). Méthodologie de description, quantification des variables morphodynamiques d'un cours d'eau à fond caillouteux: Exemple d'une station sur la Fillière (Haute-Savoie). *Revue de Géographie de Lyon, 64*(4), 252–259.

Nautiyal, P., & Mishra, A. S. (2022). Role of depth, habitat and current velocity on distribution of benthic macroinvertebrate fauna in the Himalayan River, Ramganga. *Proceedings of the Zoological Society*, *75*(3), 349-360.

Sánchez-Hernández, J., Nunn, A. D., Adams, C. E., & Amundsen, P. A. (2019). Causes and consequences of ontogenetic dietary shifts: A global synthesis using fish models. *Biological Reviews*, *94*(2), 539–554. https://doi.org/10.1111/brv.12468

Sjödin, H., Ripa, J., & Lundberg, P. (2018). Principles of niche expansion. *Proceedings of the Royal Society B: Biological Sciences*, *285*(1893), 20182603. https://doi.org/10.1098/rspb.2018.2603

Tinker, M. T., Bentall, G., & Estes, J. A. (2008). Food limitation leads to behavioral diversification and dietary specialization in sea otters. *Proceedings of the National Academy of Sciences USA*, *105*(2), 560–565. https://doi.org/10.1073/pnas.0709263105

Villsen, K., Corse, E., Archambaud-Suard, G., Héran, K., Meglécz, E., Ereskovsky, A. V., Chappaz, R., & Dubut, V. (2022). Diet metabarcoding reveals extensive dietary overlap between two benthic stream fishes (*Zingel asper* and *Cottus gobio*) and provides insights into their coexistence. *Diversity*, *14*(5), 412. https://doi.org/10.3390/d14050412

Wood, K. A., Stillman, R. A., Wheeler, D., Groves, S., Hambly, C., Speakman, J. R., ... & O'Hare, M. T. (2013). Go with the flow: Water velocity regulates herbivore foraging decisions in river catchments. *Oikos*, *122*(12), 1720-1729. https://doi.org/10.1111/j.1600-0706.2013.00592.x
